# Supplementary figures and images for: Autocrine insulin pathway signaling regulates actin dynamics in cell wound repair
Source: PLoS Genet. 2020 Dec 11;16(12):e1009186. doi: 10.1371/journal.pgen.1009186 (PMC7758051; doi:10.1371/journal.pgen.1009186)

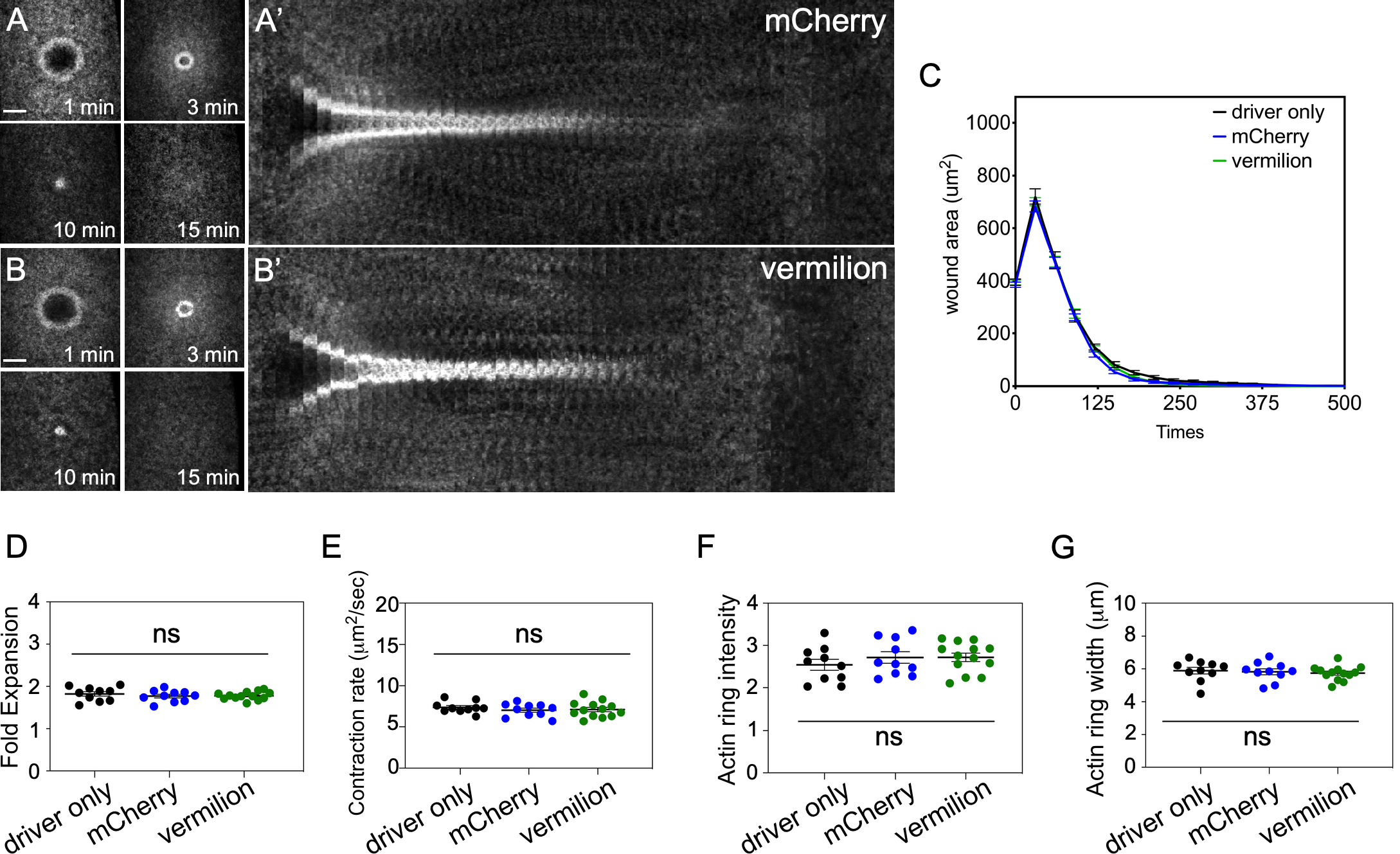

Supplement: S1 Fig — (A-B’) Confocal XY projections of actin dynamics at 1, 3, 10, and 15 mpw from Drosophila NC4-6 embryos co-expressing sGMCA with UAS-RNAi for CherryFP (A-A’) and Vermilion (B-B’) during cell wound repair. (C) Quantification of wound area over time for driver only (sGMCA, 7063/+), mCherry RNAi (sGMCA, 7063/Cherry RNAi), and vermilion RNAi (sGMCA, 7063/Vermilion RNAi). (D-G) Quantification of wound expansion (D), contraction rate (E), actin ring intensity (F), and actin ring width (G) in driver only, mCherry RNAi, and Vermilion RNAi. n ≥ 10. One way ANOVA was performed and all three pair-wise combinations were compared (driver vs mCherry RNAi, driver vs vermilion RNAi, and mCherry RNAi vs vermilion RNAi). ns = not significant. See S3 Table for numerical data. (TIF) [file pgen.1009186.s004.tif]

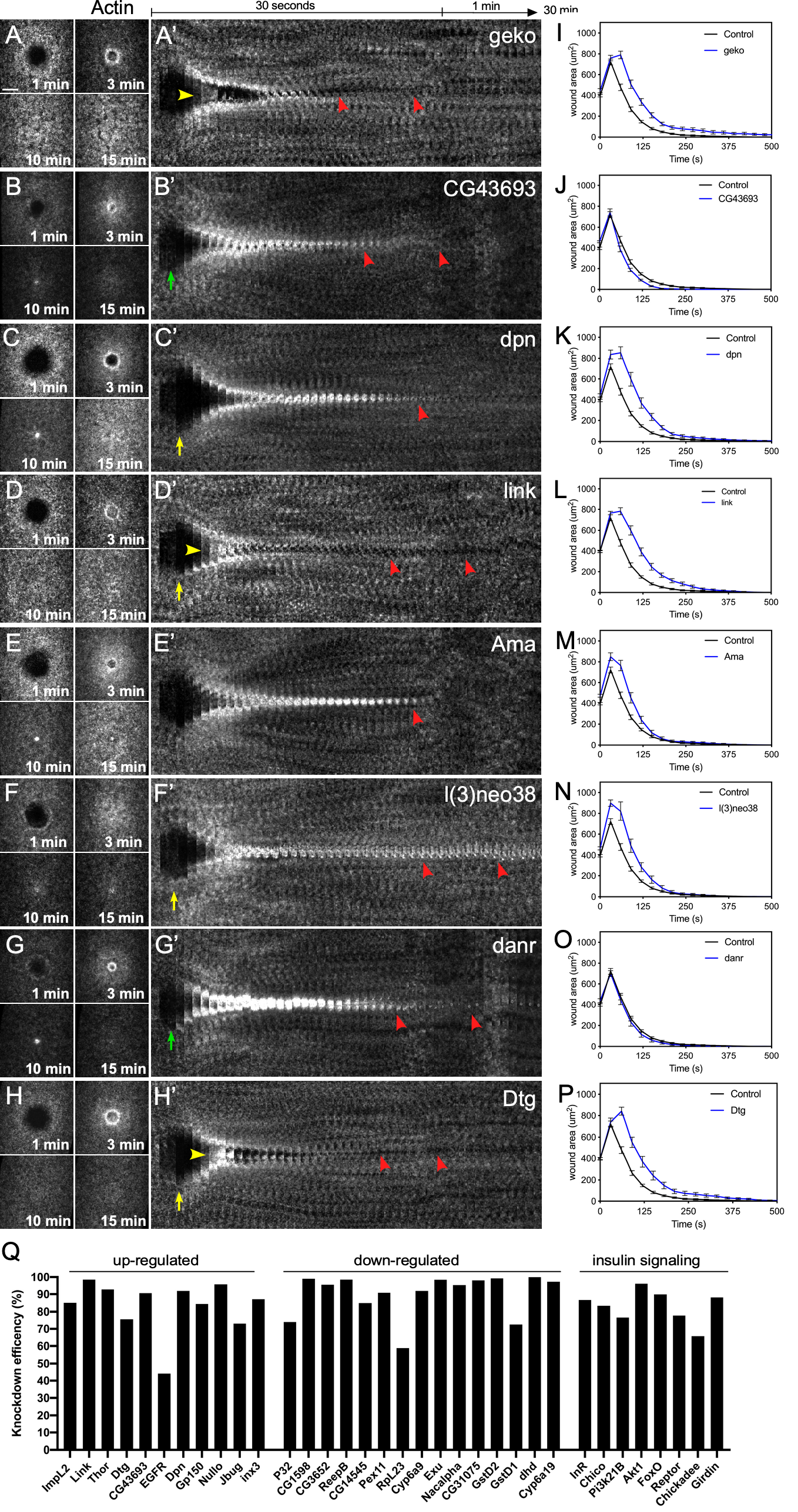

Supplement: S2 Fig — (A-H) Confocal XY projections of actin dynamics at 1, 3, 10, and 15 mpw from Drosophila NC4-6 embryos coexpressing sGMCA and a UAS-RNAi transgene during cell wound repair for GekoRNAi/+; sGMCA, 7063/+ (A), CG43693RNAi/+; sGMCA, 7063/+ (B), DpnRNAi/+; sGMCA, 7063/+ (C), LinkRNAi/sGMCA, 7063 (D), AmaRNAi/sGMCA, 7063 (E), l(3)neo38RNAi/sGMCA, 7063 (F), DanrRNAi/sGMCA, 7063 (G) and DtgRNAi/sGMCA, 7063 (H). (A’-H’) XY kymographs across the wound areas depicted in (A-H), respectively. Note wound overexpansion (yellow arrows); internal actin accumulation (yellow arrowhead), and remodeling defect/open wound (red arrowhead). (I-P) Quantification of wound area over time for (A-H’), respectively. (Q) Quantification of RNAi efficiencies for each RNAi mutant background (2 biological and 2 technical replicates were performed). Error bars represent ± SEM; n ≥ 10. See S3 Table for numerical data. Scale bars: 20 μm. (TIF) [file pgen.1009186.s005.tif]

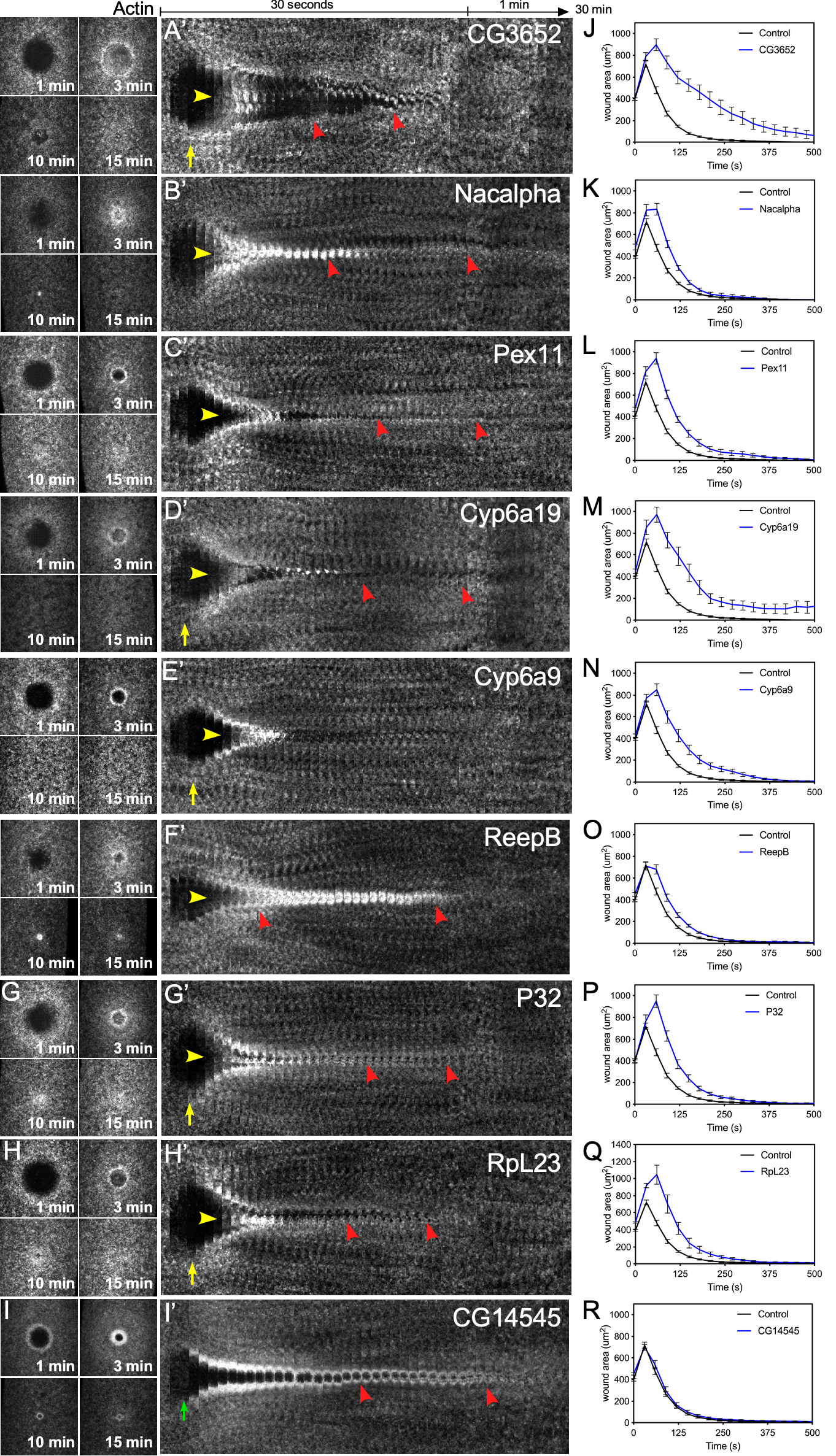

Supplement: S3 Fig — (A-I) Confocal XY projections of actin dynamics at 1, 3, 10, and 15 mpw from Drosophila NC4-6 embryos coexpressing sGMCA and a UAS-RNAi transgene during cell wound repair for CG3652RNAi/+; sGMCA, 7063/+ (A), NacAlpha RNAi/+; sGMCA, 7063/+ (B), Pex11RNAi/+; sGMCA, 7063/+ (C), Cyp6a19RNAi/sGMCA, 7063 (D), Cyp6a9RNAi/sGMCA, 7063 (E), ReepBRNAi/sGMCA, 7063 (F), P32RNAi/sGMCA, 7063 (G), RpL23RNAi/sGMCA, 7063 (H), and CG145453RNAi/sGMCA, 7063 (I). (A’-I’) XY kymographs across the wound areas depicted in (A-I), respectively. Note wound overexpansion (yellow arrows); internal actin accumulation (yellow arrowhead), and remodeling defect/open wound (red arrowhead). (J-R) Quantification of wound area over time for (A-I’), respectively. Error bars represent ± SEM; n ≥ 10. See S3 Table for numerical data. Scale bars: 20 μm. (TIF) [file pgen.1009186.s006.tif]
