# Supplementary material for: Autocrine insulin pathway signaling regulates actin dynamics in cell wound repair
Source: PLoS Genet. 2020 Dec 11;16(12):e1009186. doi: 10.1371/journal.pgen.1009186 (PMC7758051; doi:10.1371/journal.pgen.1009186)
Supplement: Attachment — Submitted filename: ResponseToReviewers_26Sep20.docx [file pgen.1009186.s012.docx]

**Response to Reviewers**

PLOS Genetics (PGENETICS-D-20-01146)

Autocrine insulin pathway signaling regulates actin dynamics in cell wound repair

M Nakamura, JM Verboon, TE Allen, MT Abreu-Blanco, R Liu, ANM Dominguez, JJ Delrow, & Susan M Parkhurst

Track Changes has been used to mark changes made to the text.

**Reviewer #1:**

**1.** *In Figure 2, the demonstrations that the inhibitors are having the expected effects on transcription and translation (currently Figs. 2H-K) should probably come before the analysis of their effects on healing (currently Figs. 2A-D).*

We take the point of the Reviewer. We have rearranged the figure (now Fig. 3) and reversed the order in which these results and controls are presented in the text.

**2.** *As noted above, deficits in single cell repair have been demonstrated for cells from diabetic mice or cells induced to become diabetic (Diabetes 2011. 60:3034-3042). This citation should be included since it strengthens the connection of the current study to other cell repair models.*

We take the point of the Reviewer and have included this study in our discussion of insulin signaling in cell wound repair (lines 287-291).

**3.** *Localization of PIP3 to vertebrate cell wounds has previously been described (Mol. Biol. Cell. 2014. 25:1867-1876). This citation should be included since it strengthens the connection of the current study to other cell repair models.*

We take the point of the Reviewer and have included this study in our discussion of PIP3 localization (lines 294-297).

**4.** *This is up to the authors, but they may wish to call attention to the fact that there is precedent for fast acting changes in translation being used to control other Rho GTPase-dependent processes, such as axon guidance (Nature 2005. 436:1020-1024).*

We now mention the precedence for fast and local translation leading to rapid regulation of cellular events (lines 372-376).

**Reviewer #2:**

**1**) *Do the RNAi lines tested in fig. 3-5 affect embryogenesis in any way?*

Some of genes tested are required for normal embryogenesis (e.g. *nullo* is required for cellularization). However, our studies are being done in very early embryos (nuclear cycle 4-10). We do not observe any defects in embryogenesis at these stages.

**2**) *What is the control in these figures? I prefer an unrelated RNAi line such as luciferase, GFP or RFP RNAi.*

The control that we include in the figures is the GAL4 driver with the actin reporter (sGMCA) in *w-* background. Since the different RNAi lines examined were not all generated in the same genetic background, we examined three controls: GAL4 driver+actin reporter (in *w-* background), GAL4 driver+actin reporter+ChFP RNAi (unrelated non-fly RNAi), and GALdriver+actin reporter+*vermillion* RNAi (unrelated fly RNAi). We find that there is no statistical difference among these three controls in any of the measured wound repair parameters. We now describe these controls in the Methods section and include a comparison of them in Supplementary Fig.1.

**3**) *Outline in more detail for the non-experts why the size of the genes is important.*

We have expanded the Discussion section describing up-regulated genes to detail the importance of identifying larger than average genes (lines 397-408).

**Reviewer #3:**

**1**) *To validate their microarray data, the authors have tested a range of parameters of healing wounds like wound area, contraction rate, actin dynamics etc in different genetic backgrounds for the duration (400s- 500s post wounding) when the post transcriptional events would be more pronounced than the transcriptional input. Thus it is not clear how much of transcriptional component of the shortlisted gene regulates healing process during this duration. In other words, down regulation of ImpL2 exhibits an altered actin dynamics right at 3 minutes post wounding, which I would believe must be because of the lower abundance of the protein at that time point and may not be directly connected with observed transcriptional up regulation 30 minutes post wounding. Secondly, can the authors delineate when the transcriptional components start playing an active role in healing process after wounding.*

It is not possible from our analyses to date to say with certainty what the contribution of transcription is to each of phenotypes examined. However, since some of genes we examined are not normally expressed in syncytial stage *Drosophila* embryos (during the timeframe of our studies), their upregulation is likely a direct effect of their response to the repair process.

**2**) *Given that Foxo and Raptor are the negative regulator of Insulin signalling, it is very difficult to comprehend the behavior of fold expansion, contraction rate, actin ring intensity of Foxo & Raptor depleted embryos with that of positive regulators of Insulin signalling like InR, dilp4, chico depleted embryos in wound healing. Since depleting both the positive and negative regulators of Insulin signalling impedes or slows the wound healing process, an explanation supported by experiments would be helpful.*

Knockdown of the different Insulin signaling components tested led to abnormal wound repair, but their phenotypes are not identical, suggesting that each downstream component of Insulin signaling has distinct roles during cell wound repair. In addition, it is not so surprising that the depletion of Foxo impedes cell wound repair as there is precedence for a role for Foxo in wound repair: Foxo is required for the prevention of oxidative stress and the depletion of Foxo impedes epithelial wound healing in keratinocytes (PMID: 24145170). There is also precedence for loss of function and overexpression of genes leading to the same phenotypes, but usually through molecular different mechanisms (cf. PMID: 3664640).

**3**) *The genes that were found to be down regulated upon wounding in the microarray data, were further down regulated using RNAi for functional analysis. I am not sure how these experiments would give meaningful insight into wound healing, however authors refer to some possibilities in the discussion section. It would be better if these hypotheses/ speculation could be supported by experiments or microarray data analysis by looking at the genes that are already implicated in wound healing so far.*

We don’t have a sufficient list of previously identified genes involved in cell wound repair that can be used for meaningful microarray data analysis as suggested. Only a small number of genes (e.g. Annexins, PKCs, RhoGEFs, RhoGAPs, E-Cadherin, and Rho family GTPases) have been identified in single cell wound repair to date. (Many more have been identified with roles in tissue/epithelial repair). Our studies are helping to remedy this issue. Indeed, as pointed out by Reviewer 1: “*The authors have exploited transcriptomic analysis of wounded Drosophila syncytial embryos to identify a wealth of candidate players in cell repair, and directly confirmed the importance of many of these using RNAi and genetic manipulations. Thus, those who study cell repair have new specific targets to investigate.”*

**4**) In Fig 6B-B'' and 6C-C'', the time elapsed post wounding is not mentioned. Going by the actin ring formation in the control samples (Fig 6B), they seems comparable to the 3 minute post wounding phenotype observed in Fig 2A and Fig 3A. If indeed they are in that timepoint, then it can be said with confidence that the transcriptional up regulation of ImpL2 clearly does not regulate the recruitment of PIP3 in the actomyosin ring region. And that would give rise to two obvious questions:

a) How is Insulin signalling aiding PIP3 recruitment on the actomyosin ring?

*b) What purpose is the observed transcriptional up regulation of ImpL2 actually serving?*

(Please note that the original Fig. 6 is now Fig.7). We are confused by the Reviewer’s comments as we did label the post-wounding time in Fig 7B (135 sec). Although we did not labeled post-wounding time in Fig 7C since we were comparing images at the same timepoint. We have now labeled the time point in both of Fig 7B and 7C to avoid confusion.

Impl2 prevents ligands binding to the receptor and PI3K act as a lipid modifier from PIP2 to PIP3 under the control of insulin signaling. The Bement lab has previously shown that *de novo* synthesized lipid domains exhibit spatial and temporal patterns during cell wound repair (PMID: 24790096). Feedback regulation from downstream pathways and/or local insulin activation by exocytosis of ligands could regulate the spatial and temporal localization of PI3K during cell wound repair, resulting in PIP3 accumulation on the actomyosin ring.

We agree that transcriptional upregulation of ImpL2 is unlikely to regulate recruitment of the PIP3 reporter upon wounding. There are two possibilities to explain the function of transcriptional up-regulation of ImpL2 during cell wound repair: since ImpL2 prevents insulin ligands from binding to the receptor, one possibility is that ImpL2 binds to wound-induced secreted ligands and then suppresses the locally activated insulin signaling to restore PIP3 levels to their unwounded state. Another possibility is that ImpL2 maintains cellular homeostasis since Insulin activation upon wounding could change the glucose and nutrient levels required for normal embryogenesis.

**5**) *Some experiments that give molecular sight into how Insulin signalling regulates actin dynamics during wound healing will be helpful for supporting the model proposed by the authors.*

We agree with the Reviewer and were interested in how insulin signaling functions to affect actin organization during cell wound repair. To that end, we included data on the localization and knockdown phenotypes of two known actin regulators, Girdin and Profilin (Fig. 9). We showed that these two proteins function under the control of insulin signaling in a spatially distinct way for proper actomyosin ring formation during cell wound repair.

**6**) *The authors propose that the Calcium surge probably causes the autocrine activation of the Insulin signalling pathway and thereby mediating the wound healing. The cited references [17, 18, 73, 74] are in either sea urchin or mammalian cell lines. The model will hold valid if the authors can also experimentally support that laser ablation causes Calcium surge, say by employing a genetically encoded calcium reporter. This might be a very important experiment to support the model. Also of interest is the source of calcium. Is calcium already present in the peri-vitelline space or is it released from the laser ablated portion of the tissue, which can be discussed? Therefore it might be important to show some degree of experimental data to hint that Calcium mediated entry might be causing the release of ILPs and thereby resulting in an autocrine IlS pathway activation. Without any substantial evidence, the inclusion of calcium in the model seems a very weak link to support the activation of IlS signalling pathway. Secondly how do the authors rule out the possibility that the laser ablation itself causes damage to the pre-packed ILPs in vesicles, thereby releasing ILPs and subsequent InR activation.*

Our laser wounding system uses a tunable laser that allows us to induce wounds at precise focal planes (i.e. the laser breaches only the cell cortex – plasma membrane and underlying cortical cytoskeleton – without damaging cell components).

We now include a new Fig.1 that shows there is a robust calcium response as measured by a calcium sensor (GCaMP6s) upon laser wounding in our Drosophila cell wound model, similar to that observed in other cell wound repair models. Although it was already shown that the perivitelline space contains calcium ions (PMID: 6402395), we examined the source of calcium using the calcium chelator BAPTA. Interestingly, we find that both intracellular and extracellular calcium stores are released upon wounding.

**7**) *The images in the panel Figure 3 F and 3H, seem to be the "exactly" same image other than the differences in contrast/brightness. I would request the authors to check it.*

We inadvertently made a copy and paste error while rearranging the order of Fig. 3 (now Fig. 4) to allow better flow of the text. We apologize for not catching this error and have corrected the affected panels.

**8**) *Have the authors attempted rescuing the phenotype of wounding by over-expressing the actin regulators, Girdin or Chickadee, in the background where Insulin signalling is down regulated ?*

Girdin and Chickadee are still expressed in the Chico knockdown background based on antibody staining – they are just not recruited to wounds (Fig. 9C-D). Thus, insulin signaling is required for the subcellular localization of Girdin and Chickadee rather than their gene expression. In addition, our results indicate that Girdin and Chickadee have distinct functions during cell wound repair. Thus, it is unlikely that overexpression of Girdin or Chickadee would rescue the phenotypes upon insulin signaling depletion.

**9**) *The discussion section may be modified to compare and contrast the present results with that of the previous ones so far including that the one reported by Kakanj et al 2016.*

The study in Kakanj et al 2016 examined the role of insulin signaling in tissue/epithelial repair rather than cell wound repair. While they wounded a single cell within the tissue, the cell was eliminated from epithelia rather than being repaired and incorporated into the epithelia. We have experience with both single cell and multicellular repair using the Drosophila models. These types of repair use different molecular mechanisms (i.e. Rho GTPase is robustly recruited to cell wounds, but is not enriched at multicellular wounds), making direct comparisons unnecessarily confusing to readers. However, as suggested by Reviewer 1, we now include discussion of cell wound repair in the diabetic state.

**10**) *I couldn't get an idea as what is exactly meant by fold expansion in the assays.*

We have previously found that wounds expand between 0 to 30 sec post-wounding and that this expansion is actively controlled during cell wound repair (PMID:21518790). Thus, measurement of wound expansion is informative as one of phenotypes in the repair process. While membrane stiffness or cytoskeletal organization could affect wound expansion, it is not yet known how wound expansion is regulated.
